# Colorectal Liver Metastases: A Literature Review of Viable Surgical Options with a Special Focus on Microwave Liver Thermal Ablation and Mini-Invasive Approach

**DOI:** 10.3390/jpm13010033

**Published:** 2022-12-23

**Authors:** Michele Finotti, Francesco Enrico D’Amico, Maurizio Romano, Marco Brizzolari, Michele Scopelliti, Giacomo Zanus

**Affiliations:** 1 4th Surgery Unit, Regional Hospital Treviso, University of Padua, DISCOG, 31100 Padua, Italy; 2 Baylor Scott & White Annette C. and Harold C. Simmons Transplant Institute, Baylor University Medical, Dallas, TX 75204, USA; 3 Second General Surgical Unit, Padova Teaching Hospital, 35128 Padua, Italy

**Keywords:** liver metastases, microwave ablation, thermal liver ablation, mini invase liver surgery, laparoscopic liver ablation

## Abstract

Colorectal cancer (CRC) is the third most common tumor worldwide and it is characterized in 20–30% of cases by liver involvement, which strongly affects the long-term patient outcome. There are many available therapies for liver colorectal metastases (CRLMs); the current standard of care is represented by liver resection, and when feasible, associated with systemic chemotherapy. Microwave thermal ablation (MWA) is a viable option in unresectable patients or to achieve treatment with a parenchymal spearing approach. A literature review was performed for studies published between January 2000 and July 2022 through a database search using PUBMED/Medline and the Cochrane Collaboration Library with the following MeSH search terms and keywords: microwave, ablation, liver metastases, colorectal neoplasm, and colon liver rectal metastases. The recurrence rate and overall patients’ survival were evaluated, showing that laparoscopic MWA is safe and effective to treat CRLMs when resection is not feasible, or a major hepatectomy in fragile patients is necessary. Considering the low morbidity of this procedure, it is a viable option to treat patients with recurrent diseases in the era of effective chemotherapy and multimodal treatments.

## 1. Introduction

### 1.1. Epidemiology and Staging

Colorectal cancer (CRC) is a major health burden; it represents 9.4% of all new tumors diagnosis and is the third most common cancer worldwide [1]. In the United States, CRC is the second cause of death related to cancer. In 2018, 130,000 new cases of colon and rectal cancer are estimated to occur, and an estimated 50,000 people will die due to colon and rectal cancer [2].

However, the incidence of colon and rectal cancers are decreasing in the last few years. It is important to note that the incidence of CRC varies over the globe, with 10-fold variability. In Australia, New Zealand, Europe, and North America for example, CRC has the highest incidence rates, while in Africa and Asia, the incidence is low. This incidence variability reflects the role of dietary and environmental on CRC development [3].

As well as the incidence, mortality is also decreasing, with the current rate of mortality of approximately 1.7 to 1.9 percent per year [4]. Cancer prevention, screening, and early diagnosis are thought to be the reason for these improvements, allowing earlier diagnosis and a wider possibility of treatments.

To note, recent data showed that in patients younger than 50 years old, for whom the standard screening tests are not recommended, the incidence is increasing. More than 1 in 10 colon cancer and 1 in 4 rectal cancers will be diagnosed in people younger than 50 years old, often with a more advanced disease [5].

The risk factors and indications for screening are well-described in the NCCN guidelines [6] for colorectal cancer screening. The population is stratified into three groups: patients with an average risk of developing CRC, patients with an increased risk, and people with a high-risk syndrome.

In addition, several environmental and lifestyle factors can contribute to an increase in the risk of CRC: obesity, diabetes mellitus, smoking, tobacco, and alcohol are most described and associated with CRC in observational studies [7,8,9,10]. 

Worldwide, the most used and preferred staging system for CRC is the TNM staging (Tumor, Node, and Metastasis that has a prognostic value) of the American Joint Committee on Cancer/Union for International Cancer Control (AJCC/UICC) [11]. The Duke’s classification and the Astler-Coller modification are not in use, currently. 

### 1.2. Prognostic Factors

Pathological staging is the most important predictor of patient outcomes. The TNM stage reflects the patient’s survival [12]. A disease stage IV (metastatic disease) has a 5-year overall survival of <5%. 

The other most influential prognostic factors are the following: lymphovascular and perineural invasion, the presence of extramural tumor deposits, histologic grade differentiation, preoperative level of serum carcinoembryonic antigen (CEA), microsatellite instability (MSI), and RAS and BRAF mutations. In Table 1, we summarize the most important prognostic factors. 

In CRC, a significant number of patients still develop metastatic disease, with an important decrease in survival. In 19–31% [1] of these patients there is a liver involvement, synchronous (found at the time of presentation of the primary tumor) or metachronous (found later). The development of liver metastases, compared to other organ involvement, is associated with a more robust decrease in the overall survival (OS): the median survival without treatment is <8 months from the moment of its presentation, and patients with local metastases can reach a survival rate at 5 years of 11% [24]. In some cases and highly selected patients, recent trials proved that liver transplantation for non-resectable CRLM is a surgical option with an excellent long-term OS [25]

This review aims to summarize the most important tools available for the treatment of colon-rectal liver metastases, with a special focus on the role of the ablation technique. 

## 2. Materials and Methods

### 2.1. Review of the Literature

A literature review was performed for studies published between January 2000 and July 2022 through a database search using PUBMED/Medline and the Cochrane Collaboration Library with the following MeSH search terms and keywords: microwave, ablation, liver metastases, colorectal neoplasm, colon liver rectal metastases. The results were completed by a manual search of references from selected reviews and papers. For the clinical data, only studies published in English with a minimum of 10 patients, with a follow-up of more than 6 months, reporting the local recurrence and/or the overall survival were included.

### 2.2. Colorectal Cancer Liver Metastases and Surgical Therapy

In presence of Colorectal Liver Metastases (CRLMs) pharmacological therapy alone is rarely a curative treatment. Surgical therapy, in particular liver resection, is an important chance for selected patients. The surgical resection, as for HCC, allows a cytoreduction of the tumor, improving the effect of natural immunological defenses and the chemotherapy that can control the microscopic metastatic disease with surgical mortality reported under 5 percent [26,27,28]. Nowadays, the gold standard to treat CRLMs is represented by systemic chemotherapy associated with liver resection. This approach shows a 5-year survival ranging from 31 to 58% [29,30]. 

Appropriate patient selection is essential to allow the best post-operative and oncologic outcomes. 

One of the most important factors that predict overall survival and the risk of recurrence is tumor biology. Based on this concept, the risk of recurrence can be stratified using four possible clinical risk scores: Fong [31], Nordlinger [32], Nagashima [33], and Konopke [34] score (see Table 2). Risk scores have limited impact on patient selection on daily bases as the curative intent of the treatment is always the goal; however, it is important to know them to help to categorize and recognize high-risk patients. 

In addition to tumor biology, anatomic factors can be a limit to liver resection. 

The definition of non-resectable liver metastases is still not clear and under evolution, with a recent more aggressive surgical approach. 

Recent guidelines and consensus define resectable as a tumor that can be resected completely with adequate margin (R0), leaving an adequate liver remnant [35]. In particular, numbers of metastases, localization, dimension, the presence of extrahepatic disease, and vascular invasion (possibility of reconstruction of vena cava and/or portal vein) should no longer restrict the indication to liver resection [36]. 

### 2.3. Non-Resectable Colorectal Liver Metastases

In almost 20–40% of patients with CRLMs, liver resection is not feasible, and the feasible half of patients will develop recurrence [37] due to localization, the number of metastasis, high operative risk, low performance status of the patient, or a combination of these factors. 

In some cases, neoadjuvant chemotherapy can allow liver resection; studies reported a percent between 12 and 33 of patients that after chemotherapy can be resected, especially with Oxaliplatin/irinotecan + fluorouracil and leucovorin protocol [38,39,40,41,42,43,44]. 

Minimally invasive techniques are other available tools, such as radiofrequency ablation (RFA) and microwave ablation (MWA) [45]. These techniques of tumor ablation provide an alternative to liver resection and can be performed in combination with systemic chemotherapy. In the last decades, locoregional treatment gained interest, especially in the contest for parenchyma-sparing disease clearance. In non-resectable liver metastases, the NCCN guidelines considered local therapies as a feasible alternative to resection, although the exact role remains controversial [6]. 

These techniques can be performed by different approaches under ultrasound or CT scan guide: percutaneous, laparoscopic, and open. 

Most clinical data on CRLM ablation are based on experience with RFA, especially with the percutaneous approach [46,47,48,49,50]. The mRECIST criteria are the current standard methods to determine the treatment efficacy, evaluating the extent of necrosis by comparing the tumor dimensions before and after the ablative treatment [51,52]. 

Recurrences after liver ablation are not uncommon and can develop in a different liver area compared to the ablation zone. In particular, local tumor progression (LTP) is defined as the persistence of active, enhancing tissue within or adjacent to the ablation site; intrasegmental recurrence (ISR) is the occurrence of CRLM nodules in the same liver segment where ablation had been performed; intrahepatic recurrence (IHR) is the appearance of CRLM nodules in other liver segments (apart from any LTP or ISR).

Usually, in most studies, to evaluate the effectiveness of the ablative technique, a computerized tomography (CT) scanner magnetic resonance imaging (MRI) of the abdomen with contrast is performed at 3, 6, and 12 months of the ablation therapy to evaluate its efficacy.

The use of MWA to treat CRLMs is less investigated despite several theoretical advantages compared to RFA (possibility to use higher temperatures, lower time of intervention, no heat-sink effect, and greater ablation volume obtainable). Some studies report a 3-year survival rate of 43–73% local tumor progression (LTP) rate of 6–51% and a complete ablation rate of 97.6% [53,54,55,56].

### 2.4. Microwave Ablation and Technical Aspects

The goal of thermal ablation is to heat malignant tissues plus a 5–10 mm margin with high temperatures to induce coagulative necrosis (typically over 60 °C). Effective treatment can be difficult to achieve in the liver where high tissue perfusion and large blood vessels can act as “heat sinks” near the ablation zone. Such heat sinks can lead to sub-lethal temperatures and sparing of malignant cells, thereby increasing the likelihood of LTP. 

RFA ablation was for a long time the most commonly used thermal ablation modality. In RFA, electrical current is passed through the tumor and adjacent tissues to generate heat; however, RFA seems to be limited by the size of the tumor and the position of the tumor, in particular in highly-vascularized tissue has made effective treatment of liver tumors over 3 cm challenging.

MWA ablation does not suffer from the same limitations. Microwave systems utilize a coaxial antenna to deliver high-frequency electromagnetic fields (915 MHz or 2.45 GHz) into the target tumor. The rapidly alternating electric field causes water and other polar molecules to rotate in an attempt to realign with the electric field. This realignment process generates kinetic energy in the tissue, raising temperatures over 100 °C and causing necrosis of tissue near the antenna. Electromagnetic fields from MWA energy are capable of continuous transmission through this desiccated and charred tissue. As a result, MWA ablation systems can create larger ablation zones than RF systems, even in the presence of large blood vessels.

MW generates temperatures above 100 °C, causing water vapor in the tissue, creating bubbles. During this time, bubbles in the ablation zone can be appreciated during ultrasound evaluation. Therefore, the MW ablations can be seen in real-time using ultrasound imaging [54]. 

### 2.5. Surgical Procedure

The surgical procedures can be performed with a laparoscopic or percutaneous approach. 

The percutaneous approach is usually preferred in case of a single nodule, elevated comorbidities of the patients, and nodules not exophytic or near hollow/vital organs (stomach, heart, duodenum, and colon).

The laparoscopic approach is preferred in case of multiple nodules, nodules not detectable with percutaneous sonographic, superficial/exophytic nodules with a high risk of postoperative bleeding, and nodules near the liver portal, arterial or biliary branch. 

### 2.6. Patient Selection and Safety Profile

CRLMs have been increasingly treated with alternative loco-regional thermal ablation, especially in patients who are poor candidates for surgery or considered unresectable. In several observational studies, complications are generally low after MWA ranging between 0 and 54% [54,55,56,57,58,59,60]. A loco-regional thermal ablation is an important tool, especially in old people with multiple comorbidities (ECOG score ≥ 1 and an ASA score ≥ 3) or patients that underwent previous liver treatments, such as liver resections and/or other liver ablations. 

The usually reported short operative time, the ability to treat more than one nodule in the same procedure, the minimal intraoperative blood loss, the rare complications after surgery, and the short length of hospital stay are all factors that represent the safety of the minimally invasive approach. 

Groeschl et al. reported 198 cases of MW ablation with the open, percutaneous, or laparoscopic approach: the median hospital stay was 1 day (range: 0–12) for percutaneous, 2 days (0–14) for laparoscopic, and 5 days (1–34) for open MWA. In the laparoscopic and percutaneous approach, a Clavien Dindo higher than II grade was reported in 9.3% and 11.1% of patients, respectively [61]. Si Qin et al. treated 411 CRLMs with only the percutaneous approach, reporting fever and pain as the most common complications. Major and minor complications occurred in 5 cases (3.65%) and 11 cases (8.03%), respectively, especially for nodules near important structures (bile duct) [60]. In the literature, the most common complication was peri- and post-procedural pain, fever, and slight pleural effusion. The most important major complications due to thermal damage to contemplate are biliary injury and bowel perforation (especially in the percutaneous approach) [62].

### 2.7. Efficacy of MWA for CRLMs and Recurrence Rate

In the literature, according to the mRECIST classification, when described, the reported success of the ablation ranges between 88% and 97% [56,61,63,64,65]. The size of the tumor was reported as the most important predictive factor of incomplete ablation and recurrence. Liu et al. stated that there was no significant difference in the complete ablation rate between MWA and RFA (93.5 vs. 84.3%). Conversely, the complete ablation rate of tumors 3.0 cm or less was significantly higher than that of tumors greater than 3.0 cm (93.5 vs. 66.7%) [63]. 

In a large series of 875 tumors treated with MWA (hepatocellular carcinoma, colorectal liver metastases, neuroendocrine liver metastases, and other cancer), ablation was successful for 97.0%. To note, in the univariate analysis, only histology was associated with incomplete ablation, whereas neuroendocrine liver metastases had greater odds of incomplete ablation compared with other histologic types. In adjusted, multivariable logistic regression no other factors were associated with incomplete ablation [61].

Alexander ES et al. in a 9-year retrospective analysis of 64 patients with single metastases (including colorectal cancer, breast cancer, carcinoid, melanoma, lung cancer, and anal cancer) who underwent MWA reported a technical success rate of 95% [66].

Most studies have been published regarding the use of RFA and MWA ablation for the treatment of hepatocellular carcinoma (HCC). However, colon rectal liver metastases have important differences compared to HCC: the role of the micrometastases. 

The size of the “ablated volume” based on the size of the primary lesion is still controversial; considering that the micrometastases can be present beyond the borders of the tumor, an appropriate safety margin of about 5 to 10 mm seems to be important to achieve complete tumor ablation, reducing the risk of local recurrence; however, the safety margin is very difficult to evaluated, especially in the follow up, considering the limitation of the CT/MRI scan and the existing intra observer variability. Recommendations about the ideal margin size to obtain during thermal ablation, without increasing the risk of complications, are currently lacking [66].

Table 3 reviewed the LTP, IHR, EHR, and the overall recurrence in studies using MWA for the treatment of CRLMs (open, laparoscopic, or percutaneous). 

Most of the studies reported a recurrence analysis of MW ablation for CRLMs using a percutaneous or an open approach. The study of Groeschl et al. is one of the largest series of laparoscopic MWA for the treatment of CRLM.

Groeschl et al., in 198 patients, reported a 5.2% of LTP and a remote intrahepatic location (>1 cm from the site of ablation) as the most common location of recurrence (26%). The frequency of local recurrence was highest after percutaneous MWA and for tumor sizes 3 cm or more [61]. 

A recent study analyzed the factors predicting ablation site recurrence after percutaneous MWA for CRLMs. Ablation site recurrence was higher in nodules near large hepatic vein (OR 7.5 95% CI 2.4–22.8) and affected by metastases size (OR 0.953 95% CI 0.929–0.978) but no association was found with the overall survival [87].

### 2.8. Overall Survival after MWA for CRLMs

MWA presents some theoretical advantages to RFA, especially proved in HCC treatment: MW ablates a larger and more uniform area than RFA; MWA is not influenced by tissue conductance, allowing better ablation results near vessels [65,88].

Table 4 summarizes the largest series of MWA used to treat CRLM reporting the OS. 

Several studies reported a 1-, 3- and 5-year OS for MWA between 40 and 98%, 40–78%, and 20–55%. Mortality was reported between 0 and 2%. The median survival ranged between 20 and 55 months, with a local recurrence between 2–25%. 

Recently, a phase II trial randomized study proved a long-term survival benefit for patients treated with RFA plus chemotherapy compared with chemotherapy alone. A total of 119 patients were recruited and treated with systemic treatment alone (FOLFOX with or without bevacizumab) or systemic treatment and RFA. With a median follow-up of 9.7 years, the OS was improved in the combined modality arm with a 3-, 5-, and 8-year OS of 56.9%, 43.1%, and 35.9% compared to 55.2%, 30.3%, and 8.9% for the chemotherapy alone arm [92].

Aukje A. et al. [93] compared MWA versus RFA for CRLM: a total of 199 lesions in 122 patients were treated with open or percutaneous MWA or RFA (48 and 151 lesions treated with MWA and RFA, respectively). With a median OS of 42 months, the study reported a comparable efficacy at 12 months of MWA and RFA, with a recurrence rate of 21.9% (33/151) for RFA treated lesions versus 39.6% (19/48) for MWA-treated lesions. Remarkably, biliary complications were especially common after peribiliary MWA 57.1% (4/7) versus RFA 3.2% (1/31).

A recent study reviewed the role of RFA and MWA compared to systemic chemotherapy and partial hepatectomy [94]. After a meta-analysis, including 48 studies and considering the OS and the complication of the procedures, Meijerink et al. concluded that thermal ablation (MWA or RFA) for small unresectable CRLM is an effective and safe tool to induce long-term disease control compared to chemotherapy alone [94]. In particular, MWA showed a median OS in five case series ranging between 24 and 36 months with a 3-, 4-, and 5-year OS between 35–79%, 35–58%, and 17–18%, respectively. The reported ablative site recurrence considering 8 series was between 2 and 30% [94].

Another meta-analysis compared MWA ablation to liver resection for the treatment of liver cancer; although the analyses include hepatocellular carcinoma and liver metastases, the study concludes that MWA can be an effective and safe alternative to liver resection in patients/tumors that are not amenable to resection [95]. Recent studies showed that MWA is effective and safe even in the case of perivascular liver metastases, achieving satisfactory margins [96]. Furthermore, a recent study compared the outcomes between open surgical resection and percutaneous microwave ablation for CRLM ≤ 3 cm showing no significant differences in OS or DFS between the two groups at 5 years. 

Recently, the European guidelines for CRLMs include ablative techniques as a first line therapy with oligometastatic disease, considering the similar outcome compared to surgical resection [97]. 

Considering the efficacy and survival benefit of MWA in the treatment of CRLMs, most of the studies showed a success rate of ablation between 88% and 97%, where size (> or <3 cm), number of nodules and the role of micrometastasis are reported to be the most important predictive factors of incomplete ablation and recurrence. When the treatment is associated with the correct chemotherapy regimen, MWA ablation is an effective tool to control the disease, especially in non-resectable diseases; however, the heterogeneity of the studies (patient selection, period, type of approach, biological features of the tumor, chemotherapy, palliative, or curative intent of the treatment) makes the comparison among them difficult. Randomized trials are needed to determine most rigorously the cause-effect relation between the MWA treatment and patient outcome.

A large multicentre, phase III, randomized controlled trial (COLLISION trial; NCT03088150) is comparing liver resection with thermal ablation (RFA or MWA) [98].

## 3. Conclusions

The use of the laparoscopic and percutaneous minimally invasive, loco-regional approach is a viable option to integrate with chemotherapy in the treatment of CRLMs. When feasible, and does not require major hepatectomies to treat small tumors, liver resection of CRLM is the treatment of choice. In the other patients, the MWA approach allows obtaining good local tumor control, associated with a low risk of peri- and post-procedural complications. In selected cases, with a median of 1 day of hospital stay in most of the studies, the patient can be treated safely and effectively, and can proceed quickly with chemotherapy when indicated [99].

Usually, the laparoscopic approach is associated with a lower LTP and better overall survival compared to the percutaneous approach. The laparoscopic approach, when feasible, better identifies the intrahepatic lesions, allows for a complete intraoperative liver ultrasound, and provides excellent control and targeting of the thermal ablation procedure. Furthermore, it allows a peritoneal exploration, a higher degree of sensitivity of intraoperative ultrasound (US) using a laparoscopic probe, and a better visualization of the target lesions (especially lesions in critical locations and/or lesions near colon, duodenum, gallbladder, diaphragm, and heart), careful hemostasis (especially in patients with coagulation disorders and/or exophytic nodules) [100,101,102].

In case of local recurrences, the MWA treatment can be repeated over time associated with a low risk of complications.

Finally, to select a patient for MWA in the multimodal treatment of CRLM, tumor dimension, type of approach (percutaneous or laparoscopic), and previous liver surgery are factors that have to be taken into account. The indication needs to be evaluated in a multidisciplinary setting, considering thermal ablation an iterative tool in combination with chemotherapy and surgical resection. 

## Figures and Tables

**Table 1:** Prognostic factor determinants of the CRC outcome.

Prognostic Factors		
Pathologic features [13,14,15,16,17,18,19,20]	Local tumor extent	Depth of tumor penetration independently influences survival
		Residual tumor after resection (R1-R2) and circumferential margin
	Regional nodes	One of the strongest predictors of outcome
		At least 12 nodes be examined histologically to accurately determine the nodal status
	Tumor regression after neoadjuvant therapy	
	Lymphovascular invasion	
	Perineural invasion	
	Histologic type, grade of differentiation, and presence of mucin	
	Tumor border	Negative predictor: irregular, infiltrating pattern of growth
	Host immune response	Positive predictor: tumor-infiltrating lymphocytes
	Peritumoral fibrosis	Negative predictor
	Microvessel density	
	Focal neuroendocrine differentiation	
	Tumor location	Positive predictor: left-sided primary tumor location
Clinical features [20,21]	Preoperative serum CEA	A higher level of CEA has a negative prognostic value. The cut-off is unclear (≥5.0 ng/mL)
	Bowel obstruction and/or perforation	
Pathologic features [22,23]	Mismatch repair deficiency	
	RAS and BRAF	RAS mutations predict a lack of efficacy for agents targeting the EGFR
	Prognostic molecular profiles	Oncotype DX Colon Cancer Assay

**Table 2:** Risk of recurrence based on risk score.

Classification	Risk Factors (Each 1 Point)	Risk Groups
Fong [31]	Disease-free interval < 12 months	Low: 0 to 2 points High: 3 to 5 points
	Number of metastases > 1	
	Preoperative CEA level > 200 ng/mL	
	Largest liver metastasis > 5 cm	
	Lymph node-positive primary tumor	
Nordlinger [32]	Age > 60	Low: 0 to 2 points Intermediate: 3 to 4 points High: 5 to 6 points
	Serosal invasion of the primary tumor (>pT3)	
	Lymph node-positive primary tumor	
	Disease-free interval < 24 months	
	Number of liver metastases > 3	
	Largest liver metastasis > 5 cm	
Nagashima [33]	Serosal invasion of the primary tumor (>pT3)	Low: 0 to 1 point Intermediate: 2 to 3 points High: ≥4 points
	Lymph node-positive primary tumor	
	Number of liver metastases ≥ 2	
	Largest liver metastasis > 5 cm	
	Resectable extrahepatic metastases	
Konopke [34]	Number of liver metastases ≥ 4	Low: 0 points Intermediate: 1 point High: ≥2 points
	CEA ≥ 200 ng/mL	
	Synchronous liver metastases	

**Table 3:** Rates of recurrence after MWA for CRLM: a review of the literature.

References	N. of Patients	Procedure	Mean *n*. of Metastases	Mean Diameter (mm)	FU (Months)	LTP (%)	IHR (%)	EHR (%)	Overall Recurrence (%)
Seki et al. [67]	15	Percutaneous	1	21	37	7			60
Shibata et al. [68]	14	Open	4.1	27	11.3				50
Liang et al. [69]	74	Percutaneous	2	31	25.1	14	51		65
Tanaka et al. [70]	16	Open	2.2	48	19	12.5	56.3	31.3	73
Kuang et al. [71]	11	Percutaneous	1.5	28	18	5			71
Martin et al. [58]	10	Open	3	25	19	10	20		
Iannitti et al. [72]	33	Percutaneous, Open, VLS	2.6	36	19	2.7	43		
Bhardwaj et al. [73]	24	Open	2.9	2	24	2	22	9.6	
Zhou et al. [74]	35	Percutaneous	1.3		5	11.3			
Wang et al. [75]	115	Percutaneous		31	28	11	12	20	
Stattner et al. [76]	28		1	10	15	3.5	17.8	42.8	
Correa-Gallego et al. [46]	67	Open	1	10	18	6			
Eng et al. [77]	33	Open	1.5		17.7	7.8			
Stattner et al. [78]	43	Open		15	15	9.3	51	51	72
Philips et al. [79]	100		2.2	28		2	5	50	
Engstrand et al. [80]	20	Open	9	27	25	25	85	55	75
Groeschl et al. [61]	198	Open (*n* = 135) VLS (*n* = 46), Percutaneus (*n* = 17)	1	20	19	5.2	26	24	
Zhou et al. [81]	295	Percutaneous	1	29	24	8.8	35.9	27.8	
Vogl et al. [82]	132	Percutaneous	2.4	18.6	28.3	6.8	9.8		
F.E. D;Amico et al. [83]	51	Percutaneous, VLS		18	18	27.4	17.6	5.8	64.7
McEachron et al. [84]	36	Percutaneous, Open, VLS	2	19	28	4.4			
Knott et al. [85]	57	Percutaneous	1	18	42	4			
McEachron et al. [86]	36	VLS	2	19	28	4.4			
Guang-Jian Liu [60]	137	Percutaneous		15.4	17.6	5.4			

Follow Up (FU), Local Tumor Progression (LTP), IntraHepatic Recurrence (IHR), ExtraHepatic Recurrence (EHR), VideoLaparoscopic (VLS).

**Table 4:** Overall Survival after MWA for CRLM: a review of the literature.

References	N of Patients	Procedure	Mean *n*. of Metastases	Mean Diameter (mm)	EHR (%)	FU Months	1 Year OS (%)	3 Year OS (%)	5 Year OS (%)	Median Survival Months
Seki et al. [67]	15	Percutaneous	1	21	0	18				24.2
Shibata et al. [68]	14	Open	4.1	27	0		71	57	14	27
Liang et al. [69]	74	Percutaneous	2	31	5	25.1	91.4	46.4		20.5
Yokoyama et al. [89]	9	Percutaneous (*n* = 6) VLS(*n* = 3)	2.8	24						
Tanaka et al. [70]	16	Open	2.2	48	5	19	80	51	17	28
Kuang et al. [71]	11	Percutaneous	1.5	28	0	17.9				
Iannitti et al. [72]	33	Percutaneous, Open, VLS	2.6	36		19				
Martin et al. [58]	10	Open	3	25		10				
Zhang et al. [90]	34	Open, Percutaneous		28			82			
Liu et al. [63]	16	Percutaneous		23						
Wang et al. [75]	115	Percutaneous		31		28	98.1	78.7		
Zhou et al. [74]	35	Percutaneous	1.3		0	5				
Lloyd et al. [56]	56	Open, VLS	2	20						
Stattner et al. [76]	28		1	10	14	15	82	45	18	
Liang et al. [91]	86	Percutaneous	2.2							
Bhardwaj et al. [73]	24	Open	2.9	2	0	48	40			29
Stattner et al. [78]	43	Open		15		15	82	40	12	28
Eng et al. [77]	33	Open	1.5			17.7				
P. Song [53]	28		1	30		55				
Engstrand et al. [80]	20	Open	9	27		25				
Philips et al. [79]	100		2.2	28						52.4
Correa-Gallego et al. [46]	67	Open	1	10		18				55
Groeschl et al. [61]	198	Open (*n* = 135) VLS (*n* = 46), Percutaneus (*n* = 17)	1	20	11	19		45	17	32.1
De Cobelli et al. [64]	19	Percutaneous, Open, VLS	1.3	13						
Zhou et al. [81]	295	Percutaneous	1	29	0	24	81.3	42.3	24.9	33
Vogl et al. [82]	132	Percutaneous	2.4	18.6	28.3	6.8	82.7	41.6		
F.E. D;Amico et al. [83]	51	Percutaneous, VLS		18	18	27.4	92.5	55.9	43.2	
Knott et al. [85]	57	Percutaneous	1	1.8		42	96	66	47	52
Guang-Jian Liu [60]	137	Percutaneous		15.4		17.6	98.1	90.6	85.9	

Follow Up (FU), ExtraHepatic Recurrence (EHR), VideoLaparoscopic (VLS).

## Data Availability

Data sharing not applicable. No new data were created or analyzed in this study. Data sharing is not applicable to this article.

## Abbreviations

CarcinoEmbryonic Antigen (CEA), Colorectal cancer (CRC), Colon Rectal Liver Metastases (CRLM), Complete Response (CR), Disease-free survival (DFS), ExtraHepatic Recurrence (EHR), HepatoCellular Carcinoma (HCC), Intensive Curative Unit (ICU), IntraHepatic Recurrence (IHR), IntraSegmental Recurrence (ISR), Local Tumor Progression (LTP), Microwave thermal ablation (MWA), Overall Survival (OS), Radiofrequency Ablation (RFA).
